# A Scoping Review of Home-Based Vestibular Rehabilitation for Benign Paroxysmal Positional Vertigo Patients

**DOI:** 10.21315/mjms-05-2025-356

**Published:** 2025-08-30

**Authors:** Hizami Mustafa, Norazreen Omar, Alia A Alghwiri, Haidzir Manaf

**Affiliations:** 1 Physiotherapy Department, Hospital Pulau Pinang, George Town, Pulau Pinang, Malaysia; 2 Physiotherapy Department, Hospital Kepala Batas, Kepala Batas, Pulau Pinang, Malaysia; 3 Department of Physiotherapy, Faculty of Rehabilitation Sciences, University of Jordan, Amman, Jordan; 4 Center for Physiotherapy Studies, Faculty of Health Sciences, Universiti Teknologi MARA Selangor, Puncak Alam Campus, Selangor, Malaysia

**Keywords:** benign paroxysmal positional vertigo, dizziness, balance, canalith repositioning manoeuvre, home-based vestibular rehabilitation

## Abstract

Benign paroxysmal positional vertigo (BPPV) is a common vestibular disorder among older adults, characterised by dizziness and imbalance caused by the displacement of otoconia into the semicircular canals. Although canalith repositioning manoeuvres are considered the standard treatment, their high recurrence rate and the need for frequent clinic visits highlight the demand for supplementary therapeutic approaches. This review examines the effectiveness of home-based vestibular rehabilitation and the associated challenges in managing patients with BPPV. A comprehensive search of databases including PubMed, Scopus, Science Direct, Web of Science, PEDro, Mendeley, and Google Scholar identified 251 records using keywords such as “BPPV” and “home-based exercise rehabilitation.” Following the screening of 228 records and full-text review of 172 articles, 17 studies met the inclusion criteria. The total sample included 541 patients with BPPV, with intervention group sizes ranging from 10 to 154 and control group sizes from 10 to 151. Intervention durations varied between 1 and 24 weeks. The findings indicate that home-based rehabilitation can reduce dizziness, improve balance and gait, lower fall risk, and enhance quality of life. Digital tools such as telephone consultations and online platforms were frequently used to support adherence and monitor exercise performance. However, the effectiveness varied across studies, with some reporting substantial benefits and others showing limited improvements. This review highlights the potential of digital technologies in enhancing home-based vestibular rehabilitation, while also emphasising the need for further research to optimise protocols and assess long-term outcomes.

## Introduction

Benign paroxysmal positional vertigo (BPPV) is one of the most common vestibular disorders, characterised by brief episodes of vertigo triggered by changes in head or body position (1, 2). It results from the displacement of otoconia—small calcium carbonate crystals—from the utricle into the semicircular canals of the inner ear (3). This displacement disrupts normal vestibular function, causing inappropriate stimulation of the vestibular system and subsequent positional vertigo (2, 4). BPPV significantly impacts quality of life, as it can lead to dizziness, imbalance, and an increased risk of falls, especially in older adults (5–7). Studies indicate that BPPV affects approximately 40% of individuals over the age of 70 who present to specialised dizziness clinics, highlighting its substantial prevalence in this population (8–10).

Vestibular rehabilitation is a well-established treatment for BPPV and other vestibular disorders (11). It is typically performed in clinical settings under the supervision of healthcare professionals, such as physical therapists (12). This exercise-based therapy promotes central nervous system adaptation and habituation to vestibular dysfunction, aiming to restore balance and reduce symptoms (13, 14). The primary objectives of vestibular rehabilitation are to enhance gaze and postural stability, relieve vertigo, and improve the performance of daily activities (15). Numerous studies have confirmed its safety and effectiveness, demonstrating significant improvements in dizziness, balance, and overall quality of life in individuals with BPPV (16, 17).

Clinic-based vestibular rehabilitation offers considerable clinical benefits but is often associated with significant economic and accessibility challenges. In 2021, global healthcare expenditures reached approximately $9.8 trillion—10.3% of the worldwide gross domestic product—yet many countries continued to struggle with achieving proportional improvements in health outcomes (18). In Malaysia, economic assessments from the same year estimated that non-communicable diseases resulted in annual economic losses of RM64.2 billion, including RM12.4 billion in healthcare costs and disability benefits and RM51.8 billion in productivity losses (19). Beyond financial burdens, limited accessibility poses a major barrier to effective treatment. Patients in rural and semi-urban areas often travel long distances to reach rehabilitation services, face inadequate public transportation, and encounter a shortage of specialised vestibular care providers (20, 21). These persistent challenges delay timely intervention, reduce adherence to rehabilitation protocols, and increase the risk of functional decline, recurrent falls, and diminished quality of life.

Currently, growing interest in home-based programmes has positioned them as a promising complementary approach to vestibular rehabilitation (22). These programmes offer several advantages, including improved accessibility, reduced healthcare costs, and increased patient autonomy and engagement (23). Home-based vestibular rehabilitation is particularly beneficial for individuals with BPPV, as it enables consistent support and facilitates the performance of repositioning manoeuvres such as the Epley and Semont manoeuvres, which are essential for symptom relief (24). A previous systematic review found that vestibular rehabilitation—including a variety of home-based exercises—significantly reduced dizziness, improved balance, and decreased disability in affected patients (25). These findings are supported by other studies showing that structured vestibular rehabilitation programmes, incorporating home-based components, lead to notable improvements in postural stability and a reduction in dizziness episodes (26).

Integrating technology-assisted approaches, particularly virtual reality and gaming applications, into vestibular rehabilitation represents a promising strategy for improving patient outcomes. Recent meta-analyses have shown that virtual reality-based programmes significantly enhance both self-reported measures and performance outcomes in patients with vestibular dysfunction, suggesting that these innovations may increase patient engagement throughout the rehabilitation process (27, 28). For instance, Kinne et al. conducted a systematic review demonstrating that home-based vestibular rehabilitation was as effective as traditional methods, indicating that the use of technology complements rather than compromises therapeutic efficacy (27). Additional studies have reported that virtual reality applications improve balance and reduce dizziness—two critical targets in vestibular rehabilitation (26, 29).

The effectiveness of vestibular rehabilitation, whether traditional or technology-assisted, can vary depending on individual patient characteristics. Research by Kim et al. (30) demonstrated that personalised rehabilitation approaches result in better outcomes, particularly for patients with more severe impairments, highlighting the importance of tailoring interventions to the specific needs of each patient. Personalisation is essential for maximising the therapeutic benefits of both conventional and technology-driven rehabilitation methods, ultimately improving patient satisfaction and adherence to treatment protocols (31, 32).

Although existing literature indicates that home-based vestibular rehabilitation may benefit individuals with BPPV, a comprehensive review of the available evidence remains lacking. This scoping review aims to synthesise current knowledge on home-based vestibular rehabilitation in individuals with BPPV, including clinical outcomes, adherence strategies, and the integration of technology-assisted approaches. By identifying key findings, evidence gaps, and future research directions, this review seeks to inform the development and implementation of effective home-based vestibular rehabilitation programmes for this population.

## Methods

### Study Design

This review protocol has been registered with the Open Science Framework (https://osf.io/3djzp). The scoping review follows a standardised methodological framework (33). The process of identifying and screening articles for inclusion was guided by the Preferred Reporting Items for Systematic Reviews and Meta-Analyses Extension for Scoping Reviews (PRISMA-ScR) (34).

### Search Strategy

A comprehensive search for relevant studies was conducted from database inception to October 2024, using major online databases including Scopus, ScienceDirect, Web of Science, PubMed, PEDro, and Mendeley. These well-established, multidisciplinary platforms index a wide range of publications and citations, particularly within the medical and health sciences. Additional records were identified through Google Scholar by systematically refining results based on specific keywords, date restrictions, thematic relevance, and source reliability, to ensure the inclusion of credible and contextually appropriate studies. Search terms were carefully selected using a thesaurus, insights from previous research, and keyword suggestions provided by the databases. The primary search terms included BPPV, home-based exercise programmes, and related strategies, with the following Boolean string applied: ((“benign paroxysmal positional vertigo” OR “BPPV”) AND (“home-based treatment” OR “home-based rehabilitation” OR “home-based program” OR “home-based exercise” OR “home-based physical exercise” OR “home-based exercise training” OR “home-based exercise therapy”)).

### Eligibility Criteria

This review included studies that met the following criteria: full-text articles published in English between 2014 and 2024; studies involving human participants aged 18–75 years diagnosed with BPPV; and those that focused on individuals with BPPV who underwent home-based vestibular rehabilitation, either as a primary or secondary outcome measure. Studies were considered regardless of their design, setting, or duration, provided they addressed home-based vestibular rehabilitation. Narrative and systematic reviews, with or without meta-analyses, were excluded.

### Study Selection and Data Extraction

Two independent reviewers screened the titles and abstracts of the identified studies to assess their eligibility. Any discrepancies were resolved through discussion. Full-text articles of the included studies were retrieved and reviewed, and relevant data were extracted, including study design, participant characteristics, intervention details, outcome measures, and key findings (35). Table 1 provides a summary of the extracted data from the selected articles, while Table 2 presents information on the effects of home-based vestibular rehabilitation on the investigated outcome measures.

### Quality Assessment

The methodological quality of the included studies was assessed using the Joanna Briggs Institute (JBI) critical appraisal checklists, which are appropriate for various study designs (36). The risk of bias in randomised controlled trials was evaluated using the Cochrane Risk of Bias tool (RoB 2) (37). Assessment results were presented in both tabular and narrative forms. Any discrepancies in scoring between reviewers or appraisal tools were resolved by consensus to ensure consistency and rigour.

### Data Synthesis and Analysis

A narrative synthesis was conducted to summarise the findings of the included studies. Results were organised according to the type of home-based vestibular rehabilitation intervention and the reported outcomes. Quantitative data, including symptom improvement and adherence rates, were presented using descriptive statistics. The feasibility and acceptability of home-based vestibular rehabilitation were discussed based on reported patient satisfaction and adherence.

## Results

**Figure 1:**
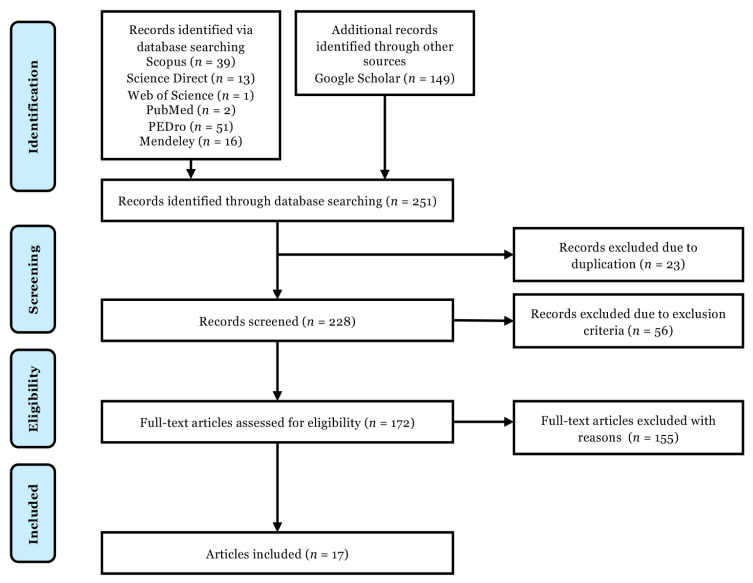
A Preferred Reporting Items for Systematic Reviews and Meta-Analysis extension for Scoping Reviews (PRISMA-ScR) flow diagram

Figure 1 presents the PRISMA-ScR flow diagram, outlining the stages of article screening and selection. The initial search identified 251 articles relevant to the topic under investigation. After screening titles and abstracts, 79 articles were excluded, leaving 172 potentially eligible publications for full-text review. Following a comprehensive evaluation of these full texts, a further 155 articles were excluded. Ultimately, 17 studies met the inclusion criteria and were selected for data extraction (8, 38–50). Table 1 summarises the key characteristics of the included studies, all of which were randomised controlled trials. In total, 541 patients with BPPV were divided into the intervention and control groups, with sample sizes ranging from 10 to 154 and 10 to 151, respectively. The duration of interventions varied from 1 to 24 weeks. Eight studies employed the Brandt-Daroff exercises as the intervention (39, 40, 42–44, 46, 51, 52), whereas seven studies implemented comprehensive home-based exercise programmes (38, 41, 45, 48–50, 53). Seven studies instructed participants to progressively increase the difficulty of exercises over time (41, 45, 47–49, 53, 54). Six studies included a supervised component—via phone, diary, or written instructions—to monitor participant adherence (41, 47–50, 53).

The JBI critical appraisal checklist for randomised controlled trials was used to evaluate the risk of bias in the included studies. The assessment revealed that six studies had a low risk of bias, whereas 11 were rated as having a moderate risk. Table 3 summarises the risk of bias evaluations. All studies employed some form of randomisation to allocate patients to their respective groups, and participants were treated identically within each trial. No crossover occurred between treatment groups, and the same assessment methods were applied to both groups. All studies followed a defined protocol for their randomised controlled trial design, and appropriate statistical analyses were conducted to support their conclusions. However, four key parameters were not consistently followed across the studies: concealment of group allocation and blinding of patients, therapists, and outcome assessors. In addition, the methodological quality of the studies was assessed using RoB 2. Bias was evaluated across the following domains: bias arising from the randomisation process, bias due to deviations from intended interventions, bias due to missing data, bias in the measurement of outcomes, and bias in the selection of the reported results. Each domain was judged as presenting a low risk, some concerns, or high risk, based on the RoB 2 algorithm.

### Exercise Protocols

Seventeen studies were reviewed to identify home-based vestibular rehabilitation protocols for individuals with BPPV. These studies commonly highlight exercise frequency, with most protocols recommending daily routines. The majority emphasise performing exercises two to three times per day—particularly exercises such as the Brandt-Daroff exercises and the Epley manoeuvre—to maintain consistency and maximise therapeutic benefit (38, 39, 41, 44, 47–54). However, some studies recommend sessions twice per week, reflecting the need for flexibility based on the clinical condition of the patient and the complexity of the intervention (43, 45).

In terms of exercise volume, seven studies specifically reported the number of repetitions or sets to be completed in each session (39, 44, 45, 48, 51, 52). Three studies recommended performing the Brandt-Daroff exercises for 5–10 repetitions, three times per day (39, 51, 52). Such details help ensure adherence to the prescribed intensity and support progression throughout rehabilitation. The volume of exercise was tailored to the specific goals of each intervention, with some protocols clearly defining sets and repetitions to align with the overall treatment plan.

The duration of each exercise session was reported in five studies, with most specifying a session length of 15 to 30 minutes (41, 45, 47, 50, 53). This timeframe enables patients to engage in progressive exercises without undue fatigue, which is essential for maintaining consistent participation and supporting recovery. For instance, studies (41, 53) recommended 15–30 minutes for gaze stabilisation and balance training exercises, whereas protocols involving more comprehensive interventions, such as yoga therapy, prescribed longer sessions of up to 45 minutes to accommodate both physical and relaxation components (50).

The types of exercises used across the studies primarily included vestibular manoeuvres, gaze stabilisation exercises, and balance training. All 17 studies reported the specific exercise interventions implemented. The Epley manoeuvre and Brandt-Daroff exercises were the most commonly prescribed for managing BPPV (39, 40, 42–46, 48, 51, 52). Gaze stabilisation exercises were also widely employed to enhance visual–vestibular integration, particularly in patients who experienced dizziness during head movements (41, 45, 47, 54). Balance training, including Cawthorne–Cooksey exercises, was frequently prescribed to improve postural control and functional mobility (41, 45, 47, 48, 54). Some studies also incorporated yoga therapy, combining balance exercises with relaxation techniques to address both physical and psychological aspects of vestibular dysfunction (49, 50).

Supervision methods were incorporated in six of the 17 studies (41, 47–50, 53). These studies employed various strategies to ensure adherence and correct execution of exercises. Remote supervision was the most commonly used approach, with telephone check-ins frequently implemented (48, 53). Some studies (41, 50) utilised WhatsApp or other digital platforms to share instructions, videos, or photographs, supporting participants in performing the exercises correctly in their home environment. Written records or exercise diaries were also used in several studies, allowing participants to log and monitor their progress—an important factor in maintaining adherence to the protocol (47, 49, 55). Moreover, some studies (41, 50) provided audio cassettes and video instructions to further aid participants in understanding how to perform the exercises properly.

### Effects of Home-Based Vestibular Rehabilitation on the Investigated Outcome Measures

Home-based vestibular rehabilitation has been extensively studied for its effectiveness in addressing dizziness, balance, gait performance, fall risk, physical activity, anxiety, and quality of life. Findings from various studies demonstrate that this approach can produce significant positive outcomes. However, the magnitude and consistency of these effects may vary depending on the specific exercise protocols used, levels of adherence, and whether additional components—such as cognitive or psychological interventions—are included.

Several studies have shown that home-based vestibular rehabilitation significantly improves dizziness and balance. Mohamad Hanapi et al. (42) reported an improvement in Dizziness Handicap Inventory scores, although specific data on physical activity or balance were not available. Similarly, Haciabbasoğlu et al. (41) observed improvements in dizziness and balance using the Dizziness Handicap Inventory and Romberg test, indicating enhanced balance and reduced dizziness. Most studies incorporating exercises such as the Brandt-Daroff exercises, vestibular rehabilitation therapy, and various forms of balance training reported notable improvements in these outcomes. For instance, Arai (38) found significant improvements in dizziness, physical activity, and quality of life among participants who engaged in combined vestibular exercises. However, some studies reported no immediate improvements in dizziness or balance, highlighting the variability in outcomes depending on the specific rehabilitation approach used (52).

Physical activity levels and gait performance are important outcomes in older adults undergoing vestibular rehabilitation. In the study by Arai (38), improvements in physical activity were observed and were associated with better physical outcomes, including increased activity volume and grip strength. Similarly, Menant et al. (53) reported significant improvements in gait performance, including reduced step-time variability and enhanced walking stability—both key indicators of mobility and fall risk. However, some studies found no direct improvements in physical activity or gait performance, despite showing benefits in dizziness and quality of life (51). This suggests that although vestibular rehabilitation may alleviate symptoms such as dizziness, its direct impact on physical activity and gait performance may be limited, particularly when physical activity is not a primary focus of the intervention.

Several studies identified a reduction in fall risk as a key benefit of home-based vestibular rehabilitation. Significant improvements in gait performance and fall risk were observed among participants receiving combined interventions (47). Assessments such as the Timed Up and Go and the Dynamic Gait Index demonstrated that home-based vestibular rehabilitation can significantly reduce fall risk by enhancing balance and gait (47). Anxiety is another important factor frequently addressed in vestibular rehabilitation programmes. Studies reported reductions in anxiety symptoms, contributing to improvements in overall quality of life (41, 49). For example, Teh et al. (49) found significant improvements in both dizziness and anxiety levels following combined vestibular exercises, suggesting that addressing both physical and psychological components in rehabilitation may enhance outcomes for individuals with vestibular disorders.

Improvements in quality of life were consistently reported across several studies, indicating that vestibular rehabilitation addresses not only physical symptoms but also psychological well-being. Significant enhancements in quality of life were observed, with vestibular exercises contributing to better overall health, increased comfort, and reduced dizziness symptoms (41, 43, 51). These findings suggest that home-based vestibular rehabilitation can effectively target both the physical and emotional dimensions of vestibular disorders, thereby enhancing overall life satisfaction.

### Adherence, Challenges, and Limitations

Adherence to home-based rehabilitation programmes varies considerably, and dropout rates may affect the generalisability of findings. For example, Jaffar et al. (43) reported no dropouts, indicating good adherence, whereas Menant et al. (53) noted the withdrawal of 44 participants, highlighting the challenges of maintaining long-term engagement. Notably, some studies also reported a small number of participants who were unable to complete the intervention or follow-up, potentially owing to the difficulty of the exercises or factors such as illness or mobility limitations (40). Adverse events associated with home-based vestibular rehabilitation were generally minimal and transient. For instance, some studies reported mild dizziness and nausea—typical and expected side effects during vestibular exercises—which were well tolerated by participants (41, 53). Overall, adverse events were rare and did not significantly affect the success of the rehabilitation programmes.

## Discussion

The evidence from the 17 reviewed studies provides a comprehensive understanding of the protocols and outcomes associated with home-based vestibular rehabilitation for patients with BPPV. This approach has been recognised as an accessible and promising option for managing BPPV, particularly in reducing residual dizziness, enhancing balance, and improving quality of life (38, 56, 57). Interventions such as the Epley manoeuvre and Brandt-Daroff exercises have shown significant benefits in alleviating dizziness and improving functional outcomes (39, 40, 42, 43). However, the findings also reveal variability in the effects of home-based vestibular rehabilitation on physical activity and gait performance, as well as challenges related to adherence and participant dropout. These factors highlight the complexities involved in delivering effective home-based care (51, 52). Although vestibular rehabilitation complements canalith repositioning manoeuvres by supporting central compensation and reducing symptom recurrence, the variation in outcomes highlights the importance of developing well-structured and individualised protocols. This is particularly crucial for older adults with comorbidities such as diabetes, hypertension, or cardiovascular disease (28), where tailored approaches are necessary to address diverse clinical needs and optimise improvements in dizziness, balance, gait performance, fall risk, physical activity, and overall quality of life (41).

The studies were qualitatively evaluated using the JBI tool for the critical appraisal of randomised controlled trials—a structured instrument designed to assess trial quality through binary responses (36). However, the JBI tool functions not merely as a checklist but as a systematic framework, as outlined in the JBI Manual for Evidence Synthesis. The risk assessment revealed low to moderate risks of bias among the included studies, with notable shortcomings in blinding. The absence of blinding for patients and therapists was considered reasonable, given that participants and providers were inherently aware of whether they received single or multiple visits. Nevertheless, blinding outcome assessors to group allocation would have helped minimise the risk of measurement bias. A key limitation across the studies was the lack of data regarding participant matching between treatment groups, introducing potential confounding variables that may have influenced the outcomes and contributed to an increased overall risk of bias.

The findings from the risk of bias assessment, conducted using RoB 2, are summarised in Table 4. The domains with the greatest proportion of high risk of bias were “bias due to deviations from intended interventions” and “bias in the measurement of outcomes,” primarily owing to the inherent difficulties in implementing blinding in interventions that require active participant involvement. In contrast, the domains with the greatest proportion of low risk of bias were “bias arising from the randomisation process” and “bias in the selection of the reported results.” These findings indicate that the methodological rigour related to random allocation and transparent outcome reporting was generally well maintained, thereby strengthening the internal validity of the included studies.

A common feature across the reviewed studies is the emphasis on daily exercise routines, with many recommending that exercises be performed two to three times per day (38, 43, 49). This frequency is particularly common in protocols involving the Brandt-Daroff exercises and the Epley manoeuvre, both designed to alleviate BPPV symptoms. The importance of repeated, consistent practice in promoting vestibular adaptation has been highlighted in several studies (39, 51). These protocols are grounded in the principle of neuroplasticity, whereby repeated exercises enhance the ability of the central nervous system to compensate for vestibular dysfunction, ultimately improving balance and reducing dizziness (58, 59). However, the need for flexibility in exercise frequency is also recognised. Studies such as those by Mohamad Hanapi et al. (42) and Teh et al. (49) support less frequent interventions, including twice-weekly sessions. This highlights the value of a personalised approach, taking into account factors such as fatigue, comorbidities, and mobility limitations, which may affect the feasibility of daily exercise regimens—particularly in frail older adults (1). As such, tailoring intervention strategies to individual needs remains key to maximising adherence and therapeutic effectiveness.

Session duration plays a crucial role in supporting patient adherence and engagement. Most studies recommend sessions lasting between 15 and 30 minutes, aligning with practical considerations related to fatigue and concentration (51). Longer sessions, such as those used in yoga-based interventions, are generally suited for individuals who benefit from a combined approach that addresses both physical and psychological needs (50). The inclusion of relaxation techniques in some protocols proves especially beneficial for older adults experiencing anxiety and stress linked to persistent dizziness and balance difficulties (49, 60). Addressing both the physical and emotional aspects of vestibular disorders remains essential for delivering comprehensive rehabilitation. As noted in previous work (61, 62), integrating cognitive-behavioural strategies can help reduce anxiety and promote overall well-being (11, 63). Rehabilitation programmes should therefore not only target physical symptoms but also incorporate psychological support to enhance patient outcomes (64).

The types of exercises prescribed across the studies primarily included vestibular manoeuvres such as the Epley manoeuvre, gaze stabilisation exercises, and balance training activities, including the Cawthorne–Cooksey exercises. These exercises are based on vestibular rehabilitation principles aimed at improving visual–vestibular integration and postural control (1). The frequent use of gaze stabilisation exercises highlights their essential role in reducing dizziness and balance deficits caused by abnormal vestibular input, particularly during head movements (65, 66). By enhancing the vestibulo-ocular reflex, these exercises help maintain stable vision during head motion while also recalibrating the sensory systems responsible for balance and spatial orientation, thereby improving postural control and lowering the risk of dizziness and falls (13, 67). Balance training exercises, often combined with vestibular manoeuvres, aim to improve functional mobility and reduce fall risk—an especially important goal in older adults, who face a higher risk of fall-related injuries (53, 59). Balance performance and postural stability improve through enhanced sensory integration, neuroplastic adaptations, and adaptive modifications, whereby the brain increases its ability to integrate vestibular, visual, and proprioceptive inputs, reorganise sensory pathways, and strengthen motor control in response to vestibular deficits (53, 59). A more comprehensive rehabilitation strategy that incorporates strengthening exercises alongside vestibular manoeuvres may offer further improvements in balance and mobility, contributing to a more holistic and effective intervention (68).

A notable innovation across the reviewed studies is the integration of remote supervision methods, which have proven effective in enhancing adherence and ensuring accurate execution of exercises. Several studies highlight the potential of remote supervision through video calls, telephone check-ins, and digital platforms such as WhatsApp (16, 69). These methods enable continuous monitoring and feedback, which is essential for ensuring correct performance of exercises in a home setting (49, 56). Furthermore, digital tools such as video demonstrations, written instructions, and exercise diaries have been shown to support patient engagement by offering both visual and written guidance—particularly beneficial for individuals who may struggle to follow verbal instructions alone (41, 47, 53). Incorporating technology into rehabilitation programmes offers a cost-effective and scalable means of delivering high-quality care, especially for populations with limited access to in-person sessions (1, 16, 23). Beyond improving access, home-based vestibular rehabilitation provides significant economic advantages that enhance its feasibility and long-term sustainability (70). By reducing the frequency of in-person visits, these programmes lower direct costs related to facility use, clinician time, and transportation. They also reduce indirect costs for patients, such as time off work, caregiver burden, and out-of-pocket expenses, particularly in rural or underserved areas. Taken together, these factors position home-based vestibular rehabilitation as a cost-effective model that supports health system efficiency while maintaining or even improving clinical outcomes.

The overall effectiveness of home-based vestibular rehabilitation in managing dizziness, restoring balance, enhancing gait performance, reducing fall risk, and improving quality of life has been widely documented. Numerous studies have reported significant reductions in dizziness, with improvements observed in objective measures such as the Dizziness Handicap Inventory and the Romberg test (41, 45, 47, 68). Reduced dizziness contributes to better balance and functional mobility, with patients reporting less disorientation during daily activities, thereby enhancing their capacity to engage in physical activity (51). However, the effects on physical activity and gait performance have been more variable. Although some studies have demonstrated improvements in activity levels and gait stability—both important for reducing fall risk (38, 53)—others, such as Gupta et al. (51), have reported limited gains in these areas. These findings suggest that although vestibular rehabilitation is effective in managing dizziness, its direct impact on mobility may be limited without complementary interventions (38, 47, 67). This variability highlights the need for a comprehensive rehabilitation approach that extends beyond symptom management to include multi-modal interventions targeting strength, endurance, and flexibility (1, 58, 71, 72).

Quality of life improvements were consistently reported across most studies, with vestibular rehabilitation contributing to better physical health, reduced dizziness-related distress, and enhanced psychological well-being. These improvements were shown to be multifaceted, addressing both the physical symptoms of vestibular disorders and their emotional consequences. For instance, several studies documented reductions in anxiety and depression levels among patients undergoing rehabilitation (41, 45, 53). This holistic approach is critical for improving overall life satisfaction, suggesting that home-based vestibular rehabilitation not only alleviates physical symptoms but also supports emotional and psychological well-being (48, 49, 63). As a scientifically grounded and accessible intervention, home-based vestibular rehabilitation optimises quality of life by promoting neuroplastic adaptation, reducing dizziness and imbalance, and enhancing functional independence. Moreover, it offers a scalable alternative to hospital-based care through personalised, professionally guided programmes (3, 64, 65).

Despite the documented benefits of home-based vestibular rehabilitation, adherence remains a significant challenge, as reflected in dropout rates reported in several studies (51–53). Contributing factors include the complexity of exercises, transient adverse effects such as dizziness or nausea, and individual patient-related issues such as comorbidities and limited social support (53, 67, 72). However, as previously discussed, remote supervision and the use of digital tools can help overcome some of these barriers by providing greater flexibility and personalised support (23, 49). Addressing these challenges requires a multidimensional approach that combines tailored patient education on exercise techniques and benefits, motivational strategies such as goal-setting and progress tracking, and individualised care plans. Such measures are essential to promote sustained engagement and maximise the effectiveness of the rehabilitation process (64).

Caregivers and family members play a vital role in supporting adherence to home-based vestibular rehabilitation, particularly among older adults and individuals with physical or cognitive limitations. Their involvement helps establish a consistent rehabilitation routine by providing supervision, encouragement, and assistance with exercises, which can reduce anxiety and build confidence in performing activities independently. Informed and engaged caregivers also contribute by reinforcing clinical goals and monitoring for symptoms or adverse effects, thereby enhancing both safety and treatment fidelity. Moreover, their participation helps create a more structured and supportive home environment that fosters sustained patient engagement. Incorporating caregiver involvement into intervention planning strengthens the real-world feasibility and long-term success of home-based rehabilitation programmes.

Adverse events during home-based vestibular rehabilitation were generally minor and short-lived, with occasional reports of side effects such as dizziness and nausea, suggesting that these protocols are safe for most participants. Such side effects are common during vestibular exercises and typically diminish as patients progress through the rehabilitation programme (1, 65). Ongoing monitoring and the provision of clear guidance on managing these symptoms can further enhance the safety and effectiveness of home-based vestibular rehabilitation.

## Conclusion

Home-based vestibular rehabilitation has emerged as a viable and effective approach for managing BPPV, particularly among older adults. The evidence presented in this review highlights the positive impact of home-based exercise programmes on a range of outcomes, including dizziness, balance, gait performance, physical activity, fall risk, and quality of life. These programmes—often incorporating exercises such as the Brandt-Daroff exercises, gaze stabilisation, and balance training—have shown significant improvements in dizziness and balance, thereby reducing fall risk. The adaptability of home-based vestibular rehabilitation, combined with remote supervision through digital tools, supports sustained patient engagement and adherence, establishing it as a highly feasible and cost-effective solution for individuals with BPPV, especially those facing mobility challenges or difficulties attending frequent clinic visits.

Despite these promising outcomes, challenges related to adherence and variability in the effectiveness of different protocols persist. Factors such as exercise complexity, comorbidities, and individual responses to treatment contribute to these variations. Although some studies report notable improvements in physical activity and gait performance, others present more heterogeneous results, suggesting that additional or complementary interventions may be needed to optimise mobility outcomes.

Furthermore, although digital technologies have enhanced the delivery and monitoring of home-based vestibular rehabilitation, further research is needed to refine exercise routines and evaluate the long-term sustainability of these interventions. Overall, the findings of this review support the continued development and implementation of tailored home-based vestibular rehabilitation programmes that address both the physical and psychological dimensions of BPPV to maximise patient outcomes. In light of the available evidence, clinicians and policymakers are encouraged to adopt comprehensive home-based vestibular rehabilitation strategies to improve accessibility, reduce disparities in care, and support consistent management across diverse populations.

## Figures and Tables

**Table 1:** Characteristics of the included studies

Author	Study design	Participant characteristics				Study period (week)	Exercise intervention	Exercise protocol				Supervision/Contact	Exercise aids
	
		Type	Sample size	Mean age (year)	Gender (M/F)			Frequency of treatment	Intensity/volume	Time per session (min)	Type/mode		
Arai (38)	RCT	NA	CG: 14	67.3 ± 7.9	0/14	24	Exercise for dizziness rehabilitation and nutritional approach	Daily	3 times/day	NA	NA	Baseline, 1st, 3rd, and 6th weeks	NA
		NA	HB: 17	62.2 ± 14.3	2/15	24	A modified Kitasato University method for dizziness rehabilitation	Daily	3 times/day	NA	NA	Baseline, 1st, 3rd, and 6th weeks	NA

Cetin et al. (39)	RCT	PCBPPV	EP: 25	51.6 ± 13.3	10/15	12–24 (Ave: 18)	Epley Manoeuvre	NA	NA	NA		Weekly	
		PCBPPV	BDE: 25	56.4 ± 11.3	11/14	12–24 (Ave: 18)	Brandt-Daroff Exercise	Daily	5 reps × 3 times/day	NA	NA	Weekly	NA

Chavan et al. (40)	Comparative study	NA	EP: 27	41.76 ± 14.25	8/17	12	Epley Manoeuvre	NA	NA	NA		Baseline, 1st week, 4th week, and 3rd month	
		NA	BDE: 27	41.48 ± 13.67	6/19	12	Brandt-Daroff Exercise	NA	NA	NA	NA	Baseline, 1st week, 4th week, and 3rd month	NA

Haciabbaso ğlu et al. (41)	Clinical trial	NA	CG: 22	51.24 ± 13.4	11/10	6	Adaptation exercises (Gaze Stabilisation)	Daily	2 times/day	NA	Not mentioned	2 times/week	WhatsApp and photographs
		NA	HB: 22	48.71 ± 8.91	7/14	6	Adaptation, balance, and movement habituation exercises	Daily	2–3 times/day	30	From easy to difficult	2 times/day	WhatsApp and photographs

Mohamad Hanapi et al. (42)	RCT	NA	SEM: 25	Not adequately reported	NA	24	Self-Epley Manoeuvre	2 weeks	NA	NA		Baseline, 1st month and 6th months	
		NA	BDE: 25	Not adequately reported	NA	24	Brandt-Daroff Exercise	2 weeks	NA	NA	NA	Baseline, 1st month and 6th months	NA

Jaffar et al. (43)	RCT	PCBPPV	CG: 10	34.10 ± 14.32	8/2	2	Brandt-Daroff Exercise	2 days/week	2 times/day	NA	NA	1st day and after second week	Not mentioned
		PCBPPV	HB: 10	29.30 ± 7.93	6/4	2	Half-Somersault Exercise	2 days/week	2 times/day	NA	NA	1st day and after second week	Not mentioned

Ravi et al. (44)	RCT	PCBPPV	CG: 47	Not specified	21/26	4	Epley manoeuvre					Baseline, 2nd week, and 1 month	
		PCBPPV	HB: 47	Not specified	22/25	4	Epley manoeuvre and Brandt-Daroff Exercise	Daily	5 reps × 2 times/day	NA	NA	Baseline, 2nd week, and 1 month	NA

Shaphe et al. (45)	RCT	PCBPPV	CG: 18	39.80 ± 3.89	6/9	4	Epley manoeuvre	2 days/week		15		Weekly	
		PCBPPV	HB: 18	39.00 ± 2.69	5/10	4	Habituation exercises, gaze stability, and balance training	2 days/week	5–10 reps	5–10	Exercises were progressed from easy to difficult	Weekly	Not reported

Sheetal et al. (46)	RCT	PCBPPV	HB: 16	45.19 ± 12.89	7/9	4	Semont Liberatory manoeuvre with Brandt-Daroff Exercise	NA	NA	NA	NA	Baseline and after 1 month	Not mentioned
		PCBPPV	CG: 14	42.79 ± 13.65	6/8	4	Brandt-Daroff Exercise	NA	NA	NA	NA	Baseline and after 1 month	Not mentioned

Smółka et al. (47)	RCT	Unilateral vestibular dysfunction	CG: 27	53.7	8/19	6	Conditioning, balance, postural, gait stability, spatial orientation training, and gaze stability exercises (at clinic)	Weekly		90	Exercises were progressed from easy to difficult	Baseline and follow-up at 6th week	Diary
		Unilateral vestibular dysfunction	HB: 31	51.94	7/24	6	Cawthorne–Cooksey exercises and simple balance exercises	Daily	2 times/day	15		Baseline and follow-up at 6th week	Diary

Taçalan et al. (48)	RCT	PCBPPV	CG: 18	46.11 ± 9.82	Not reported	6	Epley manoeuvre	During contact at the clinic				Baseline, 1st, 3rd, and 6th weeks	
		PCBPPV	HB: 18	47.93 ± 10	Not reported	6	Epley maneuver Cawthorne-Cooksey	Daily	10 reps × 2 times/day	NA	Exercises were progressed from easy to difficult	Baseline, 1st, 3rd, and 6th weeks	By telephone

Teh et al. (49)	RCT	Persistent Postural-Perceptual Dizziness	HB: 30	44.77 ± 10.04	8/22	12	MEND (Move, Eye, Neck stretching, and Deep breathing exercises) therapy	Daily	Depends on the exercise prescribed to the participants	NA	Exercises were progressed from easy to difficult	Baseline, 4 weeks, and 12 weeks	Written format
		Persistent Postural-Perceptual Dizziness	CG: 29	48.41 ± 7.33	10/19	12	Vestibular rehabilitation (customised basedon Cawthorne–Cooksey exercises)	Daily	3 times/day	NA	Not mentioned	Baseline, 4 weeks, and 12 weeks	Logbook

Vaishali et al. (50)	RCT	Chronic peripheral vertigo	HBI: 50	Not specified	20/30	12	Yoga therapy	Daily	1 time/day	30–45	Variety of yoga techniques	Baseline, 4th, 8th, and 12th weeks	Audio cassette, written record, by telephone
		Chronic peripheral vertigo	HBII: 50	Not specified	26/24	12	Vestibular exercises	Daily	2 times/day	20	Visual, proprioception, and vestibular components	Baseline, 4th, 8th, and 12th weeks	Audio cassette, written record, by telephone
		Chronic peripheral vertigo	CG: 50	Not specified	19/31	12	No exercise was prescribed					Baseline, 4th, 8th, and 12th weeks	

Gupta et al. (51)	RCT	PCBPPV	EM: 30			2	Epley manoeuvre					Baseline, 1st, and 2nd weeks	
		PCBPPV	SM: 30	49.96 ± 13.96	31/59	2	Semont Liberatory manoeuvre					Baseline, 1st, and 2nd weeks	
		PCBPPV	HB: 30			2	Brandt-Daroff Exercise	Daily	5–10 reps × 3 times/day	NA	NA	Baseline, 1st, and 2nd weeks	NA

Choi et al. (52)	RCT	PCBPPV	CG: 29	65.8 ± 8.9	8/21	1	Epley manoeuvre					Baseline and 1st week	
		PCBPPV	HB: 33	64.2 ± 12.0	8/25	1	Brandt-Daroff Exercise	Daily	10 reps × 3 times/day	NA	NA	Baseline and 1st week	NA

Menant et al. (53)	RCT	NA	CG: 151	67.6 ± 8.0	50/101	24	Usual care					Baseline and follow-up at 6th month	
		NA	HB: 154	68.0 ± 8.6	62/92	24	CRMs, home-based exercises (adaptation, substitution, habituation)	Daily	Up to 4 times/day	Up to 30	Exercises were progressed from easy to difficult	Baseline and follow-up at 6th month	Diary, by telephone

Fatima et al. (54)	RCT	Chronic dizziness	HB: 32	73.19 ± 5.53	21/11	6	Balance exercise with a gaze stability exercise	Daily	BE: 5 reps for each task/day GSE: 2 times/day	NA	Exercises were progressed from easy to difficult	Baseline, at the end of 2nd, 4th and 6th week	NA
		Chronic dizziness	CG: 32	73.0 **±** 5.69	17/15	6	Balance exercise with saccade eye exercise	Daily	BE: 5 reps for each task/day SEE: 2 times/day	NA	Exercises were progressed from easy to difficult	Baseline, at the end of 2nd, 4th and 6th week	NA

Values are presented as mean± standard deviation; RCT = randomised control trial; NA = not available; Ave = average; PCBPPV = posterior canal benign paroxysmal positional vertigo; SEM = Semont Epley maneuver; BDE = Brandt-Daroff exercise; EM = Epley maneuver; SM = Semont Liberatory maneuver; BE = balance exercise; SEE = saccade eye exercise; CG = control group; HB = home-based; reps = repetitions

**Table 2:** Effects of home-based vestibular rehabilitation on the investigated outcome measures

Author	Exercise intervention	Outcome measure							Main findings	Adherence/dropouts	Significant adverse events
		Dizziness	Physical activity	Balance stability	Gait performance	Fall risk	Anxiety	QOL			
Arai (38)	Combine	DHI	Activity volume, right and left grip strength	Gravitational, Sway Test	5 m walk speed	NA	NA	SF-8, frailty test, VAS (fatigue)	Improved dizziness, physical activity, and QOL	No dropout	NA
Cetin et al. (39)	Combine	Dix-Hallpike, VNG	NA	NA	NA	NA	NA	NA	Improved dizziness and reduced recurrence rate	Not reported	NA
Chavan et al. (40)	Combine	Dix-Hallpike, DHI	NA	NA	NA	NA	NA	NA	DHI scores improved	4 participants were unable to follow-up	NA
Haciabbaso ğlu et al. (41)	Combine	DHI, VAS	NA	Romberg Test	NA	NA	Vertigo Symptom Scale-SF, Beck Anxiety Inventory	Vertigo Dizziness Imbalance Questionnaire	Improved dizziness, balance, anxiety, and QOL	2 participants were excluded	NA
Mohamad Hanapi et al. (42)	Combine	Dix-Hallpike, DHI	NA	NA	NA	NA	NA	NA	DHI scores improved	Not reported	Well tolerated transient dizziness and nausea
Jaffar et al. (43)	Combine	NA	NA	NA	NA	Fall Efficacy Scale	NA	Vestibular Activities and Participation Measure	Improved fear of falling, residual dizziness, and QOL	No dropout	NA
Ravi et al. (44)	G2: Epley manoeuvre and Brandt-Daroff Exercise	Dix-Hallpike, VNG	NA	NA	NA	NA	NA	UCLA Dizziness Questionnaire	Improved dizziness and QOL	No dropout	NA
Shaphe et al. (45)	VRT	Dix-Hallpike	NA	BBS	NA	NA	Vertigo Symptom Scale-SF	NA	Balance, vertigo symptoms, and anxiety were improved within each group	Not receive the intervention: 3 Lost to follow-up: 2 Discontinued intervention: 1	NA
Sheetal et al. (46)	G1: Semont Liberatory manoeuvre with Brandt-Daroff Exercise	Dix-Hallpike, DHI, VAS	NA	NA	NA	NA	NA	NA	Improved dizziness	No dropout	NA
Smółka et al. (47)	Combine	DHI, VAS	NA	ALFA stabilometric platform, BBS	DGI	TUG	NA	NA	G1 significantly improves more than G2 in dizziness, balance, gait performance, and fall risk	No dropout	NA
Taçalan et al. (48)	HB	Dix-Hallpike, DHI	NA	Nintendo WBB, BBS	NA	NA	Vertigo Symptom Scale-SF	NA	Balance, vertigo symptoms, and anxiety were improved within each group	4 participants in HB were excluded	NA
Teh et al. (49)	Combine	DHI	NA	NA	NA	NA	DASS	EQ-5D	Improved dizziness, anxiety and QOL	Dropout: 1	NA
Vaishali et al. (50)	GI: Yoga therapy GII: Vestibular exercises	DHI	NA	NA	NA	NA	NA	NA	Yoga therapy was effective as vestibular exercises in improving dizziness	Adherence reviewed by telephone, dropout: 4	NA
Gupta et al. (51)	Combine	Dix-Hallpike	NA	NA	NA	NA	NA	Vestibular Activities and Participation Measure	Improved dizziness and QOL	Dropout: 17	NA
Choi et al. (52)	Combine	DHI, mSPV	NA	NA	NA	NA	NA	NA	Neither CG nor IG showed an immediate positive result	Dropout: 17	NA
Menant et al. (53)	IG	Dix-Hallpike, DHI	NA	Choice-stepping reaction time	Walking stability using step-time variability	Iconographical Falls Efficacy Scale	GAD-7 scales, PHQ-9 scale	NA	Improvement in dizziness, balance, gait performance, and fall risk was reported	Dropout: 44 participants	Adverse events were well-monitored
Fatima et al. (54)	Combine	DHI	NA	ABC Scale BBS	NA	NA	NA	NA	Balance exercises joined with gaze stability exercises, significantly improved dizziness and balance	No dropout	NA

DHI = Dizziness Handicap Inventory; BBS = Berg Balance Scale; VAS =Visual Analogue Scale; SF-8 = Short Form-8; VNG = videonystagmography; mSPV = maximal Slow Phase Velocity; QOL = quality of life; VRT = vestibular rehabilitation therapy; HB = home-based; NA = not assessed; IG = intervention group; CG = control group; G1 = group 1; G2 = group 2; m = metre

**Table 3:** The JBI Critical Appraisal Checklist for randomised controlled trials to assess the risk of bias for the included studies

Author	1. Was true randomisation used for the assignment of participants to treatment groups?	2. Was allocation to the treatment group concealed?	3. Were treatment groups similar to the baseline?	4. Were participants blind to treatment assignments?	5. Were those delivering treatment blind to treatment assignment?	6. Were outcomes assessors blind to treatment assignment?	7. Were treatment groups treated identically other than the intervention of interest?	8. Was follow-up complete and if not, were differences between groups in terms of their follow-up adequately described and analysed?	9. Were participants analysed in the groups to which they were randomised?	10. Were outcomes measured in the same way for treatment groups?	11. Were outcomes measured reliably?	12. Was appropriate statistical analysis used?	13. Was the trial design appropriate, and any deviation from the standard RCT design accounted for in the conduct and analysis of the trial?	The overall risk of bias
Arai (38)	Y	Uc	Y	Uc	Uc	Uc	Y	Y	Y	Y	Y	Y	Y	M
Cetin et al. (39)	Y	Uc	Y	Uc	Uc	Uc	Y	Y	Y	Y	Y	Y	Y	M
Chavan et al. (40)	Y	Uc	Y	Uc	Uc	Uc	Y	Y	Y	Y	Y	Y	Y	M
Haciabbasoğlu et al. (41)	Y	Uc	Y	Uc	Uc	Uc	Y	Y	Y	Y	Y	Y	Y	M
Mohamad Hanapi et al. (42)	Y	Y	Y	Y	N	N	Y	Y	Y	Y	Y	Y	Y	L
Jaffar et al. (43)	Y	Y	Y	Uc	Uc	Uc	Y	Y	Y	Y	Y	Y	Y	M
Ravi et al. (44)	Y	Uc	Y	Uc	Uc	Uc	Y	Y	Y	Y	Y	Y	Y	M
Shaphe et al. (45)	Y	Y	Y	Y	N	N	Y	Y	Y	Y	Y	Y	Y	L
Sheetal et al. (46)	Y	Uc	Y	Y	Uc	Uc	Y	Y	Y	Y	Y	Y	Y	M
Smółka et al. (47)	Y	Uc	Y	Uc	Uc	Uc	Y	Y	Y	Y	Y	Y	Y	M
Taçalan et al. (48)	Y	Y	Y	Y	N	N	Y	Y	Y	Y	Y	Y	Y	L
Teh et al. (49)	Y	Y	Y	Uc	N	N	Y	Y	Y	Y	Y	Y	Y	M
Vaishali et al. (50)	Y	Y	Y	Uc	N	Y	Y	Y	Y	Y	Y	Y	Y	L
Gupta et al. (51)	Y	Uc	Y	Uc	Uc	Uc	Y	Y	Y	Y	Y	Y	Y	M
Choi et al. (52)	Y	Uc	Y	Uc	Uc	Y	Y	Y	Y	Y	Y	Y	Y	M
Menant et al. (53)	Y	Y	Y	Y	N	N	Y	Y	Y	Y	Y	Y	Y	L
Fatima et al. (54)	Y	Y	Y	Y	Uc	Uc	Y	Y	Y	Y	Y	Y	Y	L

Y = Yes; N = No; L = Low; Uc = Unclear; M = Moderate

**Table 4:** Risk of bias assessment of included studies using the Cochrane Risk of Bias Tool 2

Author	Bias arising from the randomisation process	Bias due to deviations from intended interventions	Bias due to missing data	Bias in the measurement of outcomes	Bias in the selection of the reported result
Arai (38)	Low	High	Low	High	Some concerns
Cetin et al. (39)	Low	Some concerns	Low	Low	Low
Chavan et al. (40)	Low	Some concerns	Low	Low	Low
Haciabbasoğlu et al. (41)	Low	Some concerns	Low	Low	Low
Mohamad Hanapi et al. (42)	Low	Low	Low	Low	Low
Jaffar et al. (43)	Low	Some concerns	Low	Low	Low
Ravi et al. (44)	Low	High	Low	High	Some concerns
Shaphe et al. (45)	Low	Low	Low	Low	Low
Sheetal et al. (46)	Low	Some concerns	Low	Low	Low
Smółka et al. (47)	Low	High	Low	High	Some concerns
Taçalan et al. (48)	Low	Low	Low	Low	Low
Teh et al. (49)	Low	Some concerns	Low	Low	Low
Vaishali et al. (50)	Low	Some concerns	Low	Low	Low
Gupta et al. (51)	Low	High	Low	High	Some concerns
Choi et al. (52)	Low	High	Low	High	Some concerns
Menant et al. (53)	Low	Low	Low	Low	Low
Fatima et al. (54)	Low	Low	Low	Low	Low
